# Postoperative complications and care requirements following Achilles tendon repair: A retrospective cohort study

**DOI:** 10.1097/MD.0000000000051097

**Published:** 2026-10-23

**Authors:** Hong Zhao, Jinquan He, Weihong Yu, Rui Yang, Suhong Qi, Hongbin Cao

**Affiliations:** a Department of Foot and Ankle, Trauma Orthopedics, Tianjin Hospital, Tianjin, China; b Nursing Department, Tianjin Hospital, Tianjin, China; c The Second Ward of Foot and Ankle Department, Department of Traumatic Orthopedics, Tianjin Hospital, Tianjin, China; d The Spine 3 Ward of the Trauma and Orthopedics Department, Tianjin Hospital, Tianjin, China.

**Keywords:** Achilles tendon repair, Achilles tendon rupture, postoperative complications, risk factors, wound complications

## Abstract

Achilles tendon rupture is a common injury in orthopedic and sports medicine practice. Although surgical repair is widely performed, postoperative complications remain clinically relevant and may increase postoperative care burden. This study aimed to investigate postoperative complications and care requirements following Achilles tendon repair and to identify factors associated with postoperative complications. This single-center retrospective cohort study included patients who underwent surgical repair for acute Achilles tendon rupture between January 2021 and December 2024. Demographic characteristics, comorbidities, perioperative variables, postoperative complications, and postoperative care indicators were collected from electronic medical records. The primary outcome was the occurrence of any postoperative complication within 3 months after surgery. Secondary outcomes included length of hospital stay, number of dressing changes, outpatient follow-up visits, unplanned readmission, reoperation, and outpatient secondary wound procedures. Univariate and multivariable logistic regression analyses were performed to identify factors associated with postoperative complications. A total of 86 patients were included, and postoperative complications occurred in 16 patients (18.6%). Wound-related complications were the most common adverse events, including delayed wound healing in 6 patients (7.0%), superficial surgical site infection in 4 (4.7%), deep infection requiring operative debridement in 1 (1.2%), and chronic wound problems requiring prolonged outpatient care in 2 (2.3%). Compared with patients without complications, those with complications had significantly higher body mass index, a higher prevalence of smoking history, and a longer interval from injury to surgery. They also had significantly longer hospital stay, more dressing changes, more outpatient visits, and higher rates of readmission and reoperation. In multivariable analysis, smoking history (odds ratio 3.21, 95% confidence interval 1.07–9.61, *P* = .037) and interval from injury to surgery (odds ratio 1.36, 95% confidence interval 1.08–1.71, *P* = .010) remained independently associated with postoperative complications. Postoperative complications following Achilles tendon repair were relatively common and were predominantly wound-related. Patients who developed complications required substantially greater postoperative care. Smoking history and a longer interval from injury to surgery were independently associated with postoperative complications.

## 1. Introduction

Achilles tendon rupture is a common injury encountered in orthopedic and sports medicine practice, particularly among middle-aged and physically active individuals. As the largest and strongest tendon in the human body, the Achilles tendon is essential for walking, running, jumping, and push-off during gait. Rupture of this tendon may therefore result in substantial functional impairment, pain, gait disturbance, and temporary or prolonged restriction of daily activities, work, and sports participation. Recent epidemiological studies have shown that the incidence of Achilles tendon rupture has increased in contemporary populations, while treatment patterns have also evolved over time, making this injury an increasingly relevant clinical problem in modern practice.^[1,2]^

Both operative and nonoperative treatments are currently used for acute Achilles tendon rupture. Although nonoperative management has gained wider acceptance in selected patients, surgical repair remains commonly performed, particularly in patients with high functional demands and in those for whom restoration of tendon continuity and early rehabilitation are prioritized.^[3]^ Previous systematic reviews and meta-analyses have suggested that operative treatment may reduce rerupture risk in comparison with nonoperative treatment, although this possible benefit may be accompanied by a higher risk of other postoperative complications, particularly wound-related problems. Accordingly, treatment selection increasingly depends on individualized assessment of functional expectations, complication risk, and perioperative management strategies.^[3]^

Despite advances in operative techniques and postoperative protocols, complications after Achilles tendon repair remain clinically important. Owing to the relatively limited soft-tissue coverage and vulnerable vascularity around the Achilles tendon, wound-related complications continue to be a major concern following surgery. Reported adverse events include delayed wound healing, wound dehiscence, superficial or deep surgical site infection, sural nerve irritation, venous thromboembolic events, rerupture, and postoperative stiffness.^[4]^ In addition, differences in complication profiles may exist between open and minimally invasive techniques; recent evidence has suggested that open repair may be associated with a higher rate of wound complications and superficial infection, whereas minimally invasive techniques may be associated with sural nerve-related symptoms in some patients.^[5]^

In routine clinical practice, the success of Achilles tendon repair should not be assessed solely by rerupture rates or functional outcome scores. The postoperative care burden associated with surgical treatment is also of considerable clinical relevance. Patients who experience complications frequently require more dressing changes, additional wound surveillance, more outpatient visits, delayed rehabilitation progression, and occasionally readmission or reoperation. These events may prolong recovery, increase healthcare utilization, and negatively influence return-to-work, return-to-sport, and overall patient satisfaction.^[6]^ In addition, postoperative rehabilitation after Achilles tendon repair remains heterogeneous across studies and institutions, and this variability may further influence recovery patterns during the early postoperative period.^[7]^

Another important issue is the identification of patients at increased risk of postoperative complications. Recent studies have emphasized the role of patient- and treatment-related factors in complication development after Achilles tendon repair. Nicotine dependence has been associated with increased rates of postoperative wound disruption and infection. More recently, predictive modeling studies have attempted to establish risk calculators for complications following Achilles tendon rupture repair,^[8]^ while comparative survival analyses have suggested that factors such as elevated body mass index and delayed surgery may contribute to adverse postoperative events.^[9]^ These findings highlight the importance of perioperative risk stratification and closer monitoring in selected high-risk patients.^[10]^

Most previous studies have focused primarily on comparisons between operative and nonoperative treatment, rerupture rates, surgical techniques, and long-term functional outcomes.^[11]^ By contrast, fewer studies have comprehensively evaluated postoperative complications together with the associated postoperative care requirements in real-world clinical settings. From a practical perspective, variables such as length of hospital stay, frequency of wound care, number of outpatient follow-up visits, readmission, and reoperation may more directly reflect the actual clinical burden associated with postoperative complications. A better understanding of these issues may help clinicians improve perioperative counseling, identify high-risk individuals, and optimize postoperative management strategies.^[12]^

Therefore, the present retrospective cohort study aimed to investigate postoperative complications and care requirements following Achilles tendon repair in a real-world clinical population. Specifically, we sought to determine the incidence and pattern of postoperative complications, compare postoperative care requirements between patients with and without complications, and identify clinical factors associated with an increased risk of adverse postoperative events.

## 2. Materials and methods

### 2.1. Study design and setting

This study was approved by the Ethics Committee of Tianjin Hospital. This retrospective cohort study was conducted at a tertiary hospital. Consecutive patients who underwent surgical repair for acute Achilles tendon rupture between January 2021 and December 2024 were identified through the institutional electronic medical record system and operative database. The study protocol was approved by the Institutional Review Board of our hospital, and the requirement for written informed consent was waived because of the retrospective nature of the study.

### 2.2. Patient selection

Adult patients who underwent primary surgical repair for acute unilateral Achilles tendon rupture during the study period were screened for eligibility. The diagnosis was established on the basis of clinical presentation, physical examination, and imaging studies when necessary, including ultrasonography or magnetic resonance imaging.

Patients were included if they were 18 years of age or older, underwent primary repair within 14 days after injury, and had complete perioperative records with at least 3 months of postoperative follow-up. Patients were excluded if they had chronic Achilles tendon rupture, previous Achilles tendon surgery on the affected side, open tendon injury, concomitant fracture or major trauma involving the ipsilateral lower extremity, incomplete clinical records, or follow-up shorter than 3 months.

### 2.3. Surgical procedures and perioperative management

All procedures were performed by experienced orthopedic surgeons. The choice of surgical technique was based on rupture characteristics, local soft-tissue condition, and surgeon preference. Patients underwent either open repair or minimally invasive/percutaneous repair.

In the open repair group, the tendon was exposed through a posteromedial longitudinal incision, and end-to-end repair was performed using nonabsorbable sutures after debridement of devitalized tissue when necessary. In the minimally invasive or percutaneous group, tendon approximation was achieved through limited incisions or stab wounds using a percutaneous suture technique. Perioperative antibiotic prophylaxis was routinely administered according to institutional protocol. Tourniquet use and anesthesia type were determined by the attending surgeon and anesthesiologist.

After surgery, the ankle was immobilized in plantar flexion using a splint or cast. Routine wound inspection was performed during hospitalization and at outpatient follow-up. Progressive mobilization and rehabilitation were initiated according to tendon healing, wound condition, and surgeon preference. Partial weight-bearing was generally introduced after the initial immobilization period, and further rehabilitation progression was individualized.

### 2.4. Data collection

Clinical data were retrospectively extracted from electronic medical records, operative reports, anesthesia records, inpatient nursing notes, and outpatient follow-up documentation.

The baseline variables included age, sex, body mass index (BMI), smoking history, diabetes mellitus, hypertension, hyperlipidemia, Charlson comorbidity index, recent fluoroquinolone exposure or local corticosteroid injection, side of injury, and injury mechanism. Smoking history was abstracted from the medical record as a binary variable indicating any documented history of cigarette smoking. Because the retrospective records did not consistently distinguish current from former smoking or quantify cumulative tobacco exposure, current and former smokers could not be analyzed separately. Perioperative variables included interval from injury to surgery, surgical technique, operative time, estimated blood loss, anesthesia type, and tourniquet use. Postoperative variables included delayed wound healing, superficial surgical site infection, deep infection requiring operative debridement, chronic wound problems requiring prolonged outpatient care, sural nerve symptoms, rerupture, symptomatic deep venous thrombosis, length of hospital stay, number of postoperative dressing changes, number of outpatient visits within 3 months, unplanned readmission, reoperation, and outpatient secondary wound procedures.

### 2.5. Outcome definitions

The primary outcome was the occurrence of any postoperative complication within 3 months after surgery. Patients were categorized into complication and non-complication groups according to whether at least 1 postoperative complication was documented during follow-up.

Postoperative complications included both wound-related and non-wound-related adverse events. Delayed wound healing was defined as incomplete epithelialization or persistent wound drainage at or beyond the routine 2-week postoperative assessment, or a need for continued wound care beyond 2 weeks because the incision had not healed as expected. Superficial surgical site infection was defined as localized erythema, swelling, pain, or discharge requiring antibiotic treatment without evidence of deep tissue involvement. Deep infection requiring operative debridement was defined as infection involving the deep tissues for which operative debridement was performed. Chronic wound problems were defined as wound conditions requiring prolonged outpatient wound management beyond 6 weeks after surgery, including repeated debridement, extended negative-pressure dressing care, or bedside secondary closure. Sural nerve symptoms were defined as postoperative numbness, paresthesia, or neuropathic pain in the sural nerve distribution documented during follow-up. Rerupture was defined as a clinically and/or radiologically confirmed recurrent rupture after the index repair. Symptomatic deep venous thrombosis was defined as thromboembolic disease confirmed by vascular ultrasonography in patients with compatible symptoms.

### 2.6. Postoperative care requirements

Secondary outcomes focused on postoperative care requirements, including length of hospital stay, number of dressing changes during the postoperative period, number of outpatient follow-up visits within 3 months after surgery, unplanned readmission, reoperation, and outpatient secondary wound procedures.

### 2.7. Follow-up

Patients were routinely followed during hospitalization and in the outpatient clinic after discharge. Standard follow-up visits generally took place at approximately 2 weeks, 1 month, and 3 months after surgery, with additional visits arranged when clinically indicated. During follow-up, wound status, postoperative symptoms, complications, need for dressing care, and recovery progress were documented in the medical record. For the present study, postoperative events occurring within 3 months were included in the primary analysis, while prolonged outpatient wound care extending beyond 6 weeks was additionally recorded when present.

### 2.8. Statistical analysis

Continuous variables were assessed for normality before analysis and are presented as mean ± standard deviation or median with interquartile range, as appropriate. Categorical variables are presented as frequencies and percentages. Comparisons between the complication and non-complication groups were performed using Student *t* test or the Mann–Whitney *U* test for continuous variables and the chi-square test or Fisher exact test for categorical variables, as appropriate.

Univariate logistic regression analysis was used to assess potential factors associated with postoperative complications. Candidate variables for the multivariable model were selected on the basis of clinical relevance and univariate associations (*P* < .10). The final model included 6 covariates (BMI, smoking history, diabetes mellitus, recent fluoroquinolone exposure or local corticosteroid injection, interval from injury to surgery, and surgical technique). Because only 16 patients experienced the primary outcome, the resulting event-per-variable ratio was low; accordingly, the multivariable analysis was considered exploratory and estimates were interpreted cautiously. Odds ratios (ORs) with 95% confidence intervals (CIs) were calculated. All statistical tests were 2-sided, and a *P* value <.05 was considered statistically significant. Statistical analyses were performed using Statistical Package for the Social Sciences version 26.0 (IBM Corp.).

## 3. Results

### 3.1. Patient inclusion and baseline characteristics

Between January 2021 and December 2024, 98 patients underwent surgical repair for acute Achilles tendon rupture at our institution. After exclusion of 12 patients because of chronic rupture, incomplete medical records, or follow-up shorter than 3 months, 86 patients were included in the final analysis.

The mean age was 41.3 ± 10.8 years, and 66 patients (76.7%) were male. The mean BMI was 26.1 ± 3.5 kg/m^2^. Smoking history, diabetes mellitus, and hypertension were present in 19 (22.1%), 10 (11.6%), and 14 patients (16.3%), respectively. Six patients (7.0%) had recent fluoroquinolone exposure or local corticosteroid injection, and 11 (12.8%) had a Charlson comorbidity index ≥ 1. Open repair was performed in 56 patients (65.1%), and minimally invasive/percutaneous repair in 30 (34.9%). Additional baseline and perioperative characteristics are summarized in Table 1.

**Table 1:** Baseline and perioperative characteristics of the study cohort.

Variable	Value
Age (yr)	41.3 ± 10.8
Age range (yr)	21–68
Male, n (%)	66 (76.7)
BMI (kg/m^2^)	26.1 ± 3.5
Smoking history, n (%)	19 (22.1)
Diabetes mellitus, n (%)	10 (11.6)
Hypertension, n (%)	14 (16.3)
Hyperlipidemia, n (%)	8 (9.3)
Charlson comorbidity index ≥ 1, n (%)	11 (12.8)
Recent fluoroquinolone exposure/local steroid injection, n (%)	6 (7.0)
Right-side injury, n (%)	49 (57.0)
Sports-related injury, n (%)	57 (66.3)
Open repair, n (%)	56 (65.1)
Minimally invasive/percutaneous repair, n (%)	30 (34.9)
Interval from injury to surgery (days)	4.8 ± 2.3
Median interval from injury to surgery (IQR)	4 (3–6)
Operative time (min)	67.4 ± 15.8
Estimated blood loss (mL)	26.7 ± 12.4
General anesthesia, n (%)	51 (59.3)
Regional/spinal anesthesia, n (%)	35 (40.7)
Tourniquet used, n (%)	72 (83.7)

Values are presented as mean ± standard deviation, median (interquartile range), or number (%), as appropriate.

BMI = body mass index, IQR = interquartile range.

### 3.2. Postoperative complications

Postoperative complications occurred in 16 of 86 patients (18.6%). Wound-related complications were the most common events, including delayed wound healing in 6 patients (7.0%), superficial surgical site infection in 4 (4.7%), deep infection requiring operative debridement in 1 (1.2%), and chronic wound problems requiring prolonged outpatient care in 2 (2.3%). Sural nerve symptoms occurred in 3 patients (3.5%), rerupture in 1 (1.2%), and symptomatic deep venous thrombosis in 1 (1.2%). Because several patients experienced >1 event, the total number of complications exceeded the number of affected patients (Table 2).

**Table 2:** Distribution of postoperative complications.

Complication type	n (%)
Any postoperative complication	16 (18.6)
Delayed wound healing	6 (7.0)
Superficial surgical site infection	4 (4.7)
Deep infection requiring debridement	1 (1.2)
Chronic wound problem requiring prolonged outpatient care	2 (2.3)
Sural nerve symptoms	3 (3.5)
Rerupture	1 (1.2)
Symptomatic deep venous thrombosis	1 (1.2)

Some patients experienced >1 complication; therefore, the total number of complication events exceeded the number of affected patients.

### 3.3. Comparison between patients with and without complications

Compared with the non-complication group, patients in the complication group had a significantly higher BMI (28.4 ± 3.6 vs 25.6 ± 3.2 kg/m^2^, *P* = .006), a higher prevalence of smoking history (43.8% vs 17.1%, *P* = .021), and a longer interval from injury to surgery (6.3 ± 2.5 vs 4.4 ± 2.0 days, *P* = .004). Diabetes mellitus (25.0% vs 8.6%, *P* = .072) and recent fluoroquinolone exposure or local corticosteroid injection (18.8% vs 4.3%, *P* = .060) were more frequent in the complication group, although these differences did not reach statistical significance. No significant differences were found in age, sex, hypertension, hyperlipidemia, injury side, or injury mechanism (Table 3).

**Table 3:** Comparison of baseline characteristics between the complication and non-complication groups.

Variable	Complication group (n = 16)	Non-complication group (n = 70)	*P* value
Age (yr)	44.8 ± 10.1	40.5 ± 10.9	.146
Male, n (%)	12 (75.0)	54 (77.1)	.856
BMI (kg/m^2^)	28.4 ± 3.6	25.6 ± 3.2	.006
Smoking history, n (%)	7 (43.8)	12 (17.1)	.021
Diabetes mellitus, n (%)	4 (25.0)	6 (8.6)	.072
Hypertension, n (%)	4 (25.0)	10 (14.3)	.302
Hyperlipidemia, n (%)	2 (12.5)	6 (8.6)	.636
Charlson comorbidity index ≥ 1, n (%)	4 (25.0)	7 (10.0)	.108
Recent fluoroquinolone exposure/local steroid injection, n (%)	3 (18.8)	3 (4.3)	.060
Right-side injury, n (%)	9 (56.3)	40 (57.1)	.949
Sports-related injury, n (%)	9 (56.3)	48 (68.6)	.337

Continuous variables were compared using the independent-samples t test, and categorical variables were compared using the chi-square test or Fisher exact test, as appropriate.

BMI = body mass index.

Open repair was more common in patients with complications than in those without complications (81.3% vs 61.4%, *P* = .123). Operative time (72.8 ± 16.7 vs 66.2 ± 15.3 minutes, *P* = .112) and estimated blood loss (31.4 ± 13.1 vs 25.6 ± 12.0 mL, *P* = .096) also tended to be higher in the complication group, but no significant between-group differences were observed in these perioperative variables. Anesthesia type and tourniquet use were comparable between groups (Table 4).

**Table 4:** Comparison of perioperative characteristics between the complication and non-complication groups.

Variable	Complication group (n = 16)	Non-complication group (n = 70)	*P* value
Interval from injury to surgery (d)	6.3 ± 2.5	4.4 ± 2.0	.004
Operative time (min)	72.8 ± 16.7	66.2 ± 15.3	.112
Estimated blood loss (mL)	31.4 ± 13.1	25.6 ± 12.0	.096
Open repair, n (%)	13 (81.3)	43 (61.4)	.123
Minimally invasive/percutaneous repair, n (%)	3 (18.8)	27 (38.6)	.123
General anesthesia, n (%)	11 (68.8)	40 (57.1)	.385
Tourniquet used, n (%)	14 (87.5)	58 (82.9)	.651

Values are presented as mean ± standard deviation or number (%), as appropriate.

### 3.4. Postoperative care requirements

Patients with postoperative complications required substantially greater postoperative care. Compared with the non-complication group, the complication group had a longer hospital stay (7.4 ± 2.1 vs 5.2 ± 1.6 days, *P* < .001), more dressing changes (5.1 ± 1.8 vs 2.7 ± 1.0, *P* < .001), and more outpatient visits within 3 months (5.6 ± 1.9 vs 3.6 ± 1.2, *P* < .001). Unplanned readmission (18.8% vs 1.4%, *P* = .009), reoperation (12.5% vs 0, *P* = .010), and outpatient secondary wound procedures (12.5% vs 0, *P* = .033) were also more frequent in patients with complications (Table 5).

**Table 5:** Comparison of postoperative care requirements between the complication and non-complication groups.

Variable	Complication group (n = 16)	Non-complication group (n = 70)	*P* value
Length of stay (d)	7.4 ± 2.1	5.2 ± 1.6	<.001
Dressing changes, n	5.1 ± 1.8	2.7 ± 1.0	<.001
Outpatient visits within 3 mo, n	5.6 ± 1.9	3.6 ± 1.2	<.001
Unplanned readmission, n (%)	3 (18.8)	1 (1.4)	.009
Reoperation, n (%)	2 (12.5)	0	.010
Outpatient secondary wound procedure, n (%)	2 (12.5)	0	.033

Outpatient secondary wound procedures included bedside secondary closure or repeated clinic-based debridement/dressing interventions.

### 3.5. Risk factor analysis

In univariate analysis, higher BMI (OR 1.27, 95% CI 1.08–1.50, *P* = .005), smoking history (OR 3.77, 95% CI 1.22–11.65, *P* = .021), and longer interval from injury to surgery (OR 1.43, 95% CI 1.12–1.82, *P* = .005) were significantly associated with postoperative complications. Diabetes mellitus, recent fluoroquinolone exposure or local corticosteroid injection, greater blood loss, and open repair showed borderline associations (Table 6).

**Table 6:** Univariate analysis of factors associated with postoperative complications.

Variable	OR	95% CI	*P* value
Age	1.04	0.98–1.10	.151
Male sex	0.89	0.23–3.39	.857
BMI	1.27	1.08–1.50	.005
Smoking history	3.77	1.22–11.65	.021
Diabetes mellitus	3.56	0.95–13.38	.059
Hypertension	2.00	0.53–7.49	.304
Charlson comorbidity index ≥ 1	3.00	0.76–11.81	.117
Recent fluoroquinolone exposure/local steroid injection	5.16	0.93–28.59	.061
Interval from injury to surgery	1.43	1.12–1.82	.005
Operative time	1.03	0.99–1.07	.118
Estimated blood loss	1.04	0.99–1.08	.098
Open repair	2.72	0.72–10.29	.139

BMI = body mass index, CI = confidence interval, OR = odds ratio.

In multivariable logistic regression, smoking history (OR 3.21, 95% CI 1.07–9.61, *P* = .037) and interval from injury to surgery (OR 1.36, 95% CI 1.08–1.71, *P* = .010) remained independently associated with postoperative complications. BMI showed a borderline association (OR 1.15, 95% CI 0.99–1.33, *P* = .068), whereas diabetes mellitus, recent fluoroquinolone exposure or local corticosteroid injection, and open repair were not independently significant (Table 7). Given the limited number of complication events relative to the number of covariates, these adjusted estimates should be interpreted as exploratory.

**Table 7:** Multivariate logistic regression analysis of factors associated with postoperative complications.

Variable	OR	95% CI	*P* value
BMI	1.15	0.99–1.33	.068
Smoking history	3.21	1.07–9.61	.037
Diabetes mellitus	2.41	0.58–9.98	.226
Recent fluoroquinolone exposure/local steroid injection	3.42	0.52–22.59	.200
Interval from injury to surgery	1.36	1.08–1.71	.010
Open repair	2.01	0.51–7.90	.316

Variables entered into the multivariate model were selected on the basis of clinical relevance and univariate analysis.

BMI = body mass index, CI = confidence interval, OR = odds ratio.

## 4. Discussion

In this retrospective cohort study conducted at a tertiary hospital, postoperative complications following Achilles tendon repair occurred in 18.6% of patients and were predominantly wound-related. Delayed wound healing, superficial surgical site infection, deep infection requiring operative debridement, and chronic wound problems accounted for most of the adverse events observed in our cohort. In addition, patients who developed complications required substantially greater postoperative care, including longer hospital stays, more frequent dressing changes, more outpatient visits, and higher rates of unplanned readmission, reoperation, and outpatient secondary wound procedures. These findings indicate that postoperative complications after Achilles tendon repair should be interpreted not only in terms of frequency, but also in terms of their broader impact on recovery and healthcare utilization. Similar studies have shown that postoperative complications after Achilles tendon repair, although often infrequent in absolute terms, may meaningfully affect subsequent treatment intensity, revision risk, and healthcare cost.^[13,14]^

The predominance of wound-related morbidity in the present study is clinically consistent with prior work on operative management of acute Achilles tendon rupture. Large database and cohort studies have shown that surgical repair is generally associated with low rates of catastrophic failure, while wound-related complications, superficial infection, and postoperative soft-tissue problems remain among the most clinically relevant events after repair.^[15,16]^ Comparative studies have further suggested that minimally invasive repair may reduce wound complications relative to open surgery, although this advantage may be offset by a greater risk of temporary sural nerve symptoms in some patients.^[17,18]^ Our data showed a similar pattern, in that wound-related complications predominated, whereas open repair tended to be more common among patients who developed postoperative complications, albeit without independent significance after adjustment. This likely reflects the practical reality that open repair is often selected in more complex tears or in cases with less favorable tendon morphology, thereby introducing treatment-selection bias in retrospective cohorts.^[19,20]^

An important contribution of the present study is the explicit evaluation of postoperative care requirements. Much of the existing Achilles tendon literature has concentrated on rerupture, surgical technique, and long-term function, whereas the downstream burden of complications has been less thoroughly quantified. In our cohort, complications translated into significantly more wound care, more follow-up, and more unplanned interventions. This observation is clinically important because the burden of postoperative care may be substantial even when the initial complication is not catastrophic. Evidence from rehabilitation-focused studies suggests that postoperative recovery after Achilles tendon repair is heterogeneous, with considerable variation in rehabilitation progression, return-to-sport readiness, and longer-term recovery trajectories.^[21–23]^ Moreover, even relatively infrequent events such as postoperative deep venous thrombosis may have measurable longer-term consequences on patient outcomes.^[24]^ Taken together, these findings support a broader concept of “postoperative success,” 1 that includes not only tendon continuity and avoidance of rerupture, but also the intensity of care required to achieve recovery.

With respect to risk factors, smoking history and a longer interval from injury to surgery remained independently associated with postoperative complications in the multivariable model, while BMI showed a borderline association. These findings are biologically plausible and consistent with several published reports, but they should be interpreted as associations rather than evidence of causation. Active tobacco use has repeatedly been associated with increased postoperative wound problems and overall complications after Achilles tendon repair.^[25]^ In the present retrospective dataset, however, smoking was recorded as a binary history variable and current and former smokers could not be reliably distinguished; therefore, the observed association should not be interpreted as an estimate specific to active smoking. A longer interval from injury to surgery has also been associated with adverse postoperative events in previous studies.^[26]^ Importantly, all patients in our cohort underwent repair within 14 days, and the mean interval was relatively short. The observed association between a longer injury-to-surgery interval and complications does not demonstrate that earlier surgery would itself reduce risk. Surgical timing may be influenced by swelling and soft-tissue condition, medical optimization, referral pathways, scheduling factors, or injury complexity, each of which may also be related to postoperative outcomes. Accordingly, this finding should be regarded as hypothesis-generating and warrants confirmation in larger studies with more detailed characterization of the reasons for surgical delay. Recent risk-modeling and registry-based studies have also identified obesity, diabetes, hyperlipidemia, hypertension, and age-related factors as potentially relevant contributors to postoperative complications or revision risk, although their significance may vary by cohort and analytic strategy.^[27]^ In our study, diabetes mellitus and recent fluoroquinolone exposure or local corticosteroid injection showed trends toward increased complication risk but did not remain independently significant after adjustment, likely reflecting the relatively small number of events. Nonetheless, these variables may still be clinically informative when identifying patients who warrant closer surveillance during the early postoperative period.

Several limitations should be acknowledged. First, this was a retrospective study from a single tertiary referral center, and the results may therefore be influenced by referral bias and local practice patterns. Second, the modest sample size and limited number of complication events reduced statistical precision. Only 16 patients experienced the primary outcome, whereas 6 covariates were included in the multivariable logistic regression model, yielding a low event-per-variable ratio and a risk of model overfitting and unstable effect estimates. The adjusted results should therefore be considered exploratory and require validation in larger cohorts. Third, smoking exposure was available only as a binary history variable; current smoking, former smoking, duration of abstinence, and cumulative tobacco exposure could not be reliably distinguished, which may have obscured differences in risk between active and remote smoking exposure. Fourth, the interval from injury to surgery may be confounded by soft-tissue swelling, medical optimization, referral pathways, scheduling factors, or injury complexity; because of the observational design, the association between surgical timing and complications should not be interpreted causally. Fifth, postoperative rehabilitation was not fully standardized, and practice variation may have affected both recovery and care utilization. Sixth, longer-term functional outcomes, patient-reported outcome measures, and return-to-play data were not systematically collected. Existing literature indicates that return-to-preinjury activity after Achilles tendon rupture can be incomplete even after surgery, particularly in high-demand athletic populations. Finally, causal inferences cannot be established from this retrospective observational design. Despite these limitations, the present study provides clinically useful real-world evidence that postoperative complications after Achilles tendon repair are closely linked to increased care burden and that perioperative risk stratification remains highly relevant in routine practice.

## 5. Conclusion

In conclusion, postoperative complications following Achilles tendon repair were relatively common in this tertiary-center retrospective cohort and were predominantly wound-related. Patients who developed complications required substantially greater postoperative care, including longer hospitalization, more frequent dressing changes, more outpatient follow-up, and higher rates of unplanned readmission, reoperation, and outpatient secondary wound procedures. Smoking history and a longer interval from injury to surgery were independently associated with postoperative complications, while higher BMI showed a borderline association. The association with surgical timing should not be interpreted as evidence that earlier surgery directly reduces complication risk; rather, it warrants further investigation in studies that can account for the clinical reasons for surgical delay. These findings support careful perioperative risk stratification and close postoperative surveillance in patients undergoing Achilles tendon repair.

## Author contributions

**Conceptualization:** Hong Zhao, Jinquan He, Weihong Yu, Rui Yang, Suhong Qi, Hongbin Cao.

**Data curation:** Hong Zhao, Jinquan He, Weihong Yu, Rui Yang, Suhong Qi, Hongbin Cao.

**Formal analysis:** Hong Zhao, Jinquan He, Weihong Yu, Rui Yang, Suhong Qi, Hongbin Cao.

**Funding acquisition:** Hongbin Cao.

**Investigation:** Hong Zhao, Hongbin Cao.

**Writing – original draft:** Hong Zhao, Hongbin Cao.

**Writing – review & editing:** Hong Zhao, Hongbin Cao.
